# Cancer: What Is It? Causes and Treatment

**Published:** 1891-12

**Authors:** C. S. Reeves

**Affiliations:** Lone Grove, Texas


For Daniel's Texas Medical Journal.

### CANCER, WHAT IS IT? CAUSE AND TREATMENT.

BY C. S. REEVES, M. D., LONE GROVE, TEX.

### No. II.

THAT tuberculous deposits in the lungs, have been radically changed, and patients *cured*, by the administration of drugs, I presume, is no longer denied by any well informed physician, who keeps up with the *art* and science of medicine. The *modus operandi* of this medication is now the principal field for investigation. Here is the rock on which split some of the brightest minds of the world's history.

These things being premised, Koch's pathological micro-organisms, pathogenic microbes, bacteria, gonococci, micrococcus (Oh! for just one more big Latin word), carry us back, back to the *one-eight-millionth of a millimeter in diameter*! Now there *is* some difference, 'tis true, between the north and northwest side of a hair, but who endorses the doctrine taught in the couplet:

> "There never was a flea so small,
> But had other fleas to bite him;
> And these again have lesser fleas,
> And so on, ad infinitum."

Orthodox heredity teaches that some forty thousand of these infinitesimal beings (more or less) intermingle and mix with the *semen*, not the blood, in generation! and after the "survival of the fittest," these monstrosities begin their work of not only racing to and fro through veins and arteries, during our mortal existence, like so many fish in the ocean; but in their everlasting metamorphoses, they actually *make blood;* or what is nearly equivalent, they compose the material of which it is made, and so it comes to pass, "that the dust of Alexander may be made to stop a bung-hole at last;" neither is it "to reason too curiously, to reason so." *Ex animo*; I lay aside all such theories as both unphilosophic and unreasonable, wholly impracticable, and withal, calculated to mislead the medical student from the path of duty. If the physiologist proves an entire change in the animal economy every 7, or 14 years for that matter; if vacination against small-pox protects the system for a lifetime against the ravages of that disease; I respectfully ask by what system of reasoning do we conclude that these bacteria are not thrown off with other effete matter? Is it not more philosophic to conclude that *cancer* (the crab) as such, is at first purely a *local disease*, that certain peculiar diatheses *invite* from without, the onslaught or attack of these cancer producing bacteria, *i. e.*, admitting as fact, that *this is* the *cause*, and *not* the *effect* of this disease?

My conclusion, from the premises already adduced, is that if the ten thousand cases of septicæmia, or blood poisoning that occur all over the country every day are successfully treated by the administration of antiseptics, there can be no valid reason against a similar mode in the treatment of the much dreaded cancer.

At this point my attention is directed to the question of Dr. M. M. Gilbert, of Arizona, in the last number of the JOURNAL. He wants to know the treatment for "screw worms in the nose."

Well, *mirabile dictu!* I have been called to treat half a dozen cases within the last four years, of screw worms in the nose, throat, ears and other localities in the *human* body! Chloroform, administered through a small syringe, and brought immediately in contact with these vermin, will prove *a dead shot* every time. A year ago I was called to attend a lady, from whose nose and *throat* were expelled no less than a pint. The time consumed in getting them away was more than ten days, caused by trying many different remedies before chloroform. Now I again ask the question, if the use of this remedy exterminated these bacteria (?) in the shape of the screw worm, and did produce no evil results otherwise, why may not a tonic blood purifier be applied in case of cancer, that will kill these bacteria? Evidently, if the doctrine of modern bacteriology be true, there can be no such thing as a *blood* purifier in medicine, only on this principle. In my next I will speak of local remedies in connection with these tonics, with which I have cured many cases called cancer by the highest medical authority in Texas. Still, I never advertise in a newspaper, nor publish a certificate of cure.

